# Second international congress on immunopharmacology: delivery systems and current strategies for drug design

**DOI:** 10.1186/1471-2172-14-S1-S1

**Published:** 2013-02-25

**Authors:** Reinaldo Acevedo, Mario Landys, Armando Acosta, Herve Bercovier, Mohd Nor Norazmi, Valerie Ferro, Maria Elena Sarmiento

**Affiliations:** 1 Finlay Institute. Ave. 27 No. 19805, La Lisa. La Habana, Cuba. AP. 16017, CP11600; 2 Faculty of Medicine, Hebrew University of Jerusalem, Israel; 3 Schools of Health Sciences Universiti Sains Malaysia, 16150 Kubang Kerian, Kelantan, Malaysia; 4 Institute for Research in Molecular Medicine, Universiti Sains Malaysia, 16150 Kubang Kerian, Kelantan, Malaysia; 5 University of Strathclyde, Strathclyde Institute of Pharmacy and Biomedical Sciences (SIPBS), Glasgow, Scotland, UK

## 

The 2^nd^ International Congress on Immunopharmacology was held in June of 2011 at the Conference Center of Plaza America in Varadero, Cuba. The main goal of this meeting was to provide state-of-the-art communications for scientists, manufacturers, regulators and healthcare workers, to accelerate progress in the development of biological and biotechnological products and to promote exchange/scientific cooperation between researchers. 300 delegates from 22 countries attended the conference. The wide-ranging programme commenced with a plenary session and then split into a series of parallel workshops and symposia, covering “Advances in Immunopharmacology”, “Neuroimmunology”, “Therapeutic Biological Products”, “Prophylaxis and Treatment of *Helicobacter pylori*”, “Pharmacology of Cytochrome P450”, “Hereditary Ataxias” and “Delivery Systems and Current Strategies for Drug Design”. In this last Symposium, a substantial body of data was presented relating to the development of delivery systems with adjuvant and vaccine potential and also to strategies focused in therapeutic and prophylactic approaches against tuberculosis. This issue is dedicated to some of the results presented in this area.

Particulated structures have been used for more than two decades in the formulation of vaccine candidates, even before nanotechnology became a common field on its own. Virus-like particles and outer membrane vesicles (OMV) based vaccines were shown to have prophylactic potential against various infections [1]. Soluble antigens obtained through recombinant or synthetic processes have been known to be less immunogenic than antigens associated with nanoparticles [2]. Traditional inactivated and attenuated whole vaccines have immunostimulatory components, like LPS and DNA, which also account for toxic reactions associated to such vaccines. However, these molecules, which are also referred to as natural adjuvants [3], may trigger signals to activate cellular pathways that potentiate the immune response to antigens in the vaccine formulation [3]. Development of bacterial derived nano/microparticles takes advantage of such immunostimulatory effects of the antigenic repertoire expressed in the outer membrane of microorganisms [4]. OMV vaccines against *Neisseria meningitidis* serogroup B were developed as both adjuvant [4] and vaccine [1]. Furthermore, OMV and cochleates obtained from bacteria have been effectively used via the mucosal route to induce systemic as well as mucosal immune responses [5]. This and other approaches have promoted research and development of novel particulated structures from *Vibrio cholerae*, *Bordetella pertussis*, *N. meningitidis* and Mycobacteria [6,7]. Presentations related with these areas are included in this supplement. Research related with the identification of antigens of *M. tuberculosis* with vaccine potential using *in silico* methods as well as work related with potential markers of tuberculosis infection are also included.

The potential importance of the specific antibody response against tuberculosis is a subject of growing interest [8-14] and a report on the protective role of antibody formulations against mycobacteria is also presented.

We are very grateful to BMC Immunology for agreeing to publish this group of presentations of the symposium.

## Declarations

This article has been published as part of *BMC Immunology* Volume 14 Supplement 1, 2013: Proceedings from Delivery Systems and Current strategies to drug design. The full contents of the supplement are available online at http://www.biomedcentral.com/bmcimmunol/supplements/14/S1
